# Diabetes patient’s pharmacovigilance knowledge and risk perception: the influence of being part of a patient organisation

**DOI:** 10.1177/2042098620953935

**Published:** 2020-09-04

**Authors:** Cristiano Matos, Florence van Hunsel, Rogério Tavares Ribeiro, Dulce Nascimento do Ó, João Filipe Raposo

**Affiliations:** Escola Superior de Tecnologia da Saúde de Coimbra – Coimbra Health School, Rua 5 de Outubro, Coimbra, 3046-854, Portugal; Department of Pharmacology, Faculty of Pharmacy, University of Seville, Seville, Spain; Netherlands Pharmacovigilance Centre Lareb,‘s-Hertogenbosch, the Netherlands; Portuguese Diabetes Association - APDP Diabetes, Lisbon, Portugal; Portuguese Diabetes Association - APDP Diabetes, Lisbon, Portugal; Portuguese Diabetes Association - APDP Diabetes, Lisbon, Portugal

**Keywords:** adverse drug reactions, diabetes, patients’ organisation, risk perception

## Abstract

**Objective:**

The aim was to assess the perception of risk for developing adverse drug reaction (ADRs) and knowledge, attitudes and opinions regarding pharmacovigilance in diabetic patients, and to investigate the effect of being a member of a patient organisation for diabetes on these factors, in comparison with other patients.

**Methods:**

A cross-sectional study looking for patients’ risk perception of experiencing ADRs. Diabetes patients followed at the Portuguese Diabetes Association (APDP) were included, together with two comparison groups (patients with and without diabetes). Kruskal-Wallis followed by *post hoc* Dunn’s multiple-comparison test were used to compare patients’ groups.

**Results:**

A total of 314 patients participated in the survey (104 followed at APDP, 106 with diabetes not followed at APDP and 104 without diabetes diagnosis that used chronic medication). APDP patients presented higher risk perception scores for medicines related to their disease compared with two groups. Those patients affirmed that doctors explained possible ADRs on medication to them, and showed higher intention to report ADRs in the future if serious or unexpected.

**Conclusions:**

Patients with diabetes showed greater understanding of ADRs and higher need to report them than patients without diabetes. They would like to have more information about general ADRs related to anti-diabetic medication and present higher intention to acquire information on how and when to report compared with non-diabetic patients. Patients followed in APDP presented higher score of risk perception, which could be influenced by the presence of the diabetes disease in the patients’ life, by their previous experiences using medicines, but also by information received from the patient organisation. The two groups of patients with diabetes have different experiences of the disease, but both present higher perception of side effects related with medicines they use respectively in their diabetes type. Hence, patient organisations are well positioned to be a source where patients can obtain reliable information, changing their attitudes and perceptions about the disease and drug treatments.

## Introduction

Risk perception can be defined as the belief (whether rational or irrational) held by an individual, group, or society about the chance of occurrence of a risk or about the extent, magnitude and timing of its effect(s).^1^ Several factors could influence general risk perception, such as the cognitive and emotional state of the perceiver, as well as subconscious and socio-cultural, economic and individual factors.^2,3^

Several studies have tried to identify the risk perception for adverse drug reactions (ADRs) in different healthcare professionals (HCPs) and patients.^4–7^ The term ‘ADR risk perception’ means that the perceiver understands the general concept that drugs have side effects or that a specific ADR is related with a medicine. Major differences were found in risk perception of ADRs between patients and HCPs,^8^ revealing the importance of adequate communication on drugs for the general population.^4,5^ Different experiences from patients and HCPs related to the use of medicines and their positive or negative views about its safety enrich their knowledge of drugs. Personal experiences with ADRs have shown to increase the perceived risk that a specific reaction will occur in the future.^9^ Moreover, there is a close relationship between the intensity of potential ADRs related to medicines and a higher risk perception for a reaction to that medicine. A significant underestimation of risks of medicines most commonly used, such as non-steroidal anti-inflammatory drugs (NSAIDS), antihypertensive drugs and oral contraceptives, has been described for both patients and HCPs.^10,11^ Previous studies have investigated general risk perception in random groups of patients,^12,13^ or evaluated risk perception for specific drug classes such as NSAIDs or paracetamol.^14–16^ Recently, risk perception in diabetes patients who have experienced adverse reactions was also studied.^9^

Risk perception and knowledge on ADRs are closely tied to pharmacovigilance, which depends highly on the adequate reporting of ADRs in a post-marketing setting.^17^ However, since underreporting of ADRs is still evident, creating awareness and promotion of ADR reporting is needed for both HCPs and patients.^18^ One of the reasons for the lack of reporting by HCPs and patients is the shared uncertainty in evaluating and recognizing an ADR.^19,20^ A low and weak risk perception, together with insufficient attention given to the training of HCPs regarding the practical approaches of pharmacology, pharmacovigilance and drug safety-related issues, are given as underlying reasons.^6,8,21^ From the patient’ point of view, underreporting can be attributed to patient-related factors like failure to recognize ADRs, inability to link the ADR to a drug, low or inexistent risk perception about newly marketed drugs or to insufficient knowledge to identify ADRs.^22,23^ Furthermore, for pharmacovigilance centers, it is important to investigate how risks are perceived and how patients could be optimally informed about drug safety issues and, subsequently, how to create a reporting culture among patients where they share their experiences with ADRs.

From the literature, it is known that patients usually underrate the risk of adverse reactions of their medications.^20^ Therefore, they should receive enough information regarding the medicines they use, including benefits and risks, as well as the correct way of administering them. However, studies have shown that this communication is virtually nonexistent or very limited.^24^ Obstacles in communication to patients could influence risk perception.^8,23,25–27^ Patients desire to be informed of ADRs related to their medication, regardless of how uncommon they are.^5^ HCPs have an important role to play in providing this information and in creating awareness of potential ADRs, since they might be prevented if patients are better informed and attentive to their risks.^28–30^ Despite this, drug risk perception is complex, and depends on the personal impression of drug users.^8^

Quantifying risk perception, knowledge of pharmacovigilance, and description of patient attitudes could identify several factors to be optimized through educational interventions, to improve knowledge about drug safety issues and, in addition, increase the reporting of ADRs for different groups of patients.^31^ Recently, there has been a lot of attention on patient-reported outcomes and initiatives to involve patients.^32–34^ Patient reporting is seen as a useful source of information in addition to HCP reporting, not just in gaining more reports, but in receiving the patient’s perspective on ADRs experienced.^35–38^ Collaboration between patient organisations and national competent authorities is important to raise awareness about pharmacovigilance among the general public,^39^ and can play a role in educating patients on risks related to medicine use and help involve them in pharmacovigilance.

Patient organisations may be pivotal in the education of patients, and their members may be better informed on the risks related to drug use.^32,40^ Members of patient organisations are usually better informed about their health and are potentially more likely to report ADRs. Patient organisations offer easy access to target groups to spread information, in an easy way, to a specific group of consumers.^38,41^ Accordingly to the European Medicines Agency (EMA), “patients’ organisations” are defined as not-for-profit organisations which are patient-focused, and whereby patients and/or carers (the latter when patients are unable to represent themselves) represent a majority of members in governing bodies”.^42^ Besides, these organisations could contribute to the reporting of ADRs to pharmacovigilance centers.^37,43–45^ The roles that patient organisations have played in supporting research and raising public awareness were highlighted in several publications.^46–51^ A recent study reveals that there is a wide range of interest in drug safety issues among patient organisations. Many organisations thought that patients could contribute to pharmacovigilance in different ways, including providing different information to that given by HCPs; aiding detection of new ADRs, their severity, and their impact on quality of life; and reporting information that was useful and based on their experience with medicines, even without medical confirmation.^39^ An important number of organisations do not appear to have activities or involvement relating to pharmacovigilance. Bringing pharmacovigilance stakeholders and patient organisations together could create a more optimal patient reporting culture.^39^ The importance of the perspective of patients on public health and the impact of regulatory decisions is clear and national competent authorities have made an effort in recent years to involve patient organisations in decision making.^52–53^

A study investigating the risk perception of medicines in general, found that patients have a lower risk perception for medicines used in diabetes (such as oral antidiabetics and/or insulin), in relation to other medicine groups, such as sleeping pills, tranquillisers or antidepressants.^5^ A recent study suggests that the personal experience of diabetes patients with adverse reactions should be considered when authorities seek to include patients in developing regulatory decisions.^9^ Although risk perception in people with diabetes has been studied previously, the role that patient organisations can play in risk perception for medicines used in diabetes remains unexplored.

The objective of this study was to assess risk perception for developing ADRs, and the knowledge, attitudes and opinions regarding pharmacovigilance in patients with diabetes, and to investigate whether being a member of a patient organisation for diabetes has an effect on risk perception, knowledge, attitudes and opinions, in comparison with other patients, both with and without diabetes.

## Methods

A cross-sectional study was conducted to investigate patients’ risk perception of experience adverse reactions related to their medication and to assess their knowledge, attitudes and opinions regarding pharmacovigilance, using face-to-face questionnaires. Respondents were recruited based on a cross-sectional design, comparing patients from a patient organisation for diabetes – the Portuguese Diabetes Association (APDP-Diabetes Portugal) – followed in their clinic with another two groups of patients, one constituted by people with diabetes not followed in the organisation and other group of patients without diabetes. APDP is a patient organisation recognised by the International Diabetes Federation as a reference centre for diabetes and receives the most serious cases in Portugal, with 40% of patients being treated with insulin. APDP provides medical support to its members through medical follow up, counselling and care (nursing and nutrition, among others).^54^

### Study population

The first group was recruited during May 2018 at the outpatient clinic of APDP, using convenience sampling; patients were included if they had a diabetes diagnosis and were over 18 years old. Only type 1 and type 2 patients were invited to participate in the study, on the days of their medical appointments, by personal interview while waiting for consultation. No gestational diabetes patients were surveyed.

Two comparison groups were recruited in a community pharmacy in Coimbra, Portugal, during June and July 2018. Comparison groups included a group of patients with diabetes not being followed at APDP and a group of patients without a diabetes diagnosis. Patients for the comparison groups were invited to participate by community pharmacy visitors. To improve comparability between groups, age and level of education distribution of the first group were taken into consideration when recruiting comparison groups. Comparison group of patients without diabetes were composed mostly of patients with chronic diseases using chronic medication. From the pharmacy records, it was possible to conclude that at least 78.8% (*n* = 82) of these patients have used medicines for a chronic disease for a period longer than 6 months. Chronic diseases included mostly heart disease, hypertension, hypercholesterolemia or respiratory diseases, among others.

Written informed consent was obtained from each participant. The Ethics Committee of APDP-Diabetes Portugal approved this study, and no financial benefits were given to the participants. The individual questionnaire was anonymous, and the data were intended only for the scientific purposes of this study and were stored in agreement with privacy regulations.

### Questionnaire design

A questionnaire for the assessment of ADR risk perception was developed by the authors in collaboration with HCPs from the APDP clinical research team. The questionnaire was pre-tested for content validity by several volunteers (*n* = 16), not included in the sample, in order to improve the understanding of the questionnaire. The content validity was assessed and discussed in the section *Strengths and limitations of the study*.

The final questionnaire included four major sections: Section I – respondents’ characteristics (gender, age, education, time since diagnosis and diabetes type). In Section II, 12 sentences were used to assess the attitudes and opinions about spontaneous reporting of ADRs, based on previous studies.^18,43,55^ Likert scales were used, and responses were coded as an ordinal scale: 1, 2, 3, 4 and 5 (representing strongly disagree, disagree, neutral, agree and strongly agree, respectively). This section also contained questions regarding experiences and knowledge on pharmacovigilance. In Section III, participants were asked to assess their perception of the risk of occurrence of any ADRs related with pre-specified drug classes, using visual analogue scales (VAS). In Section IV, only patients with diabetes were asked about the risk for ADRs associated with diabetes drugs used by them.

In the questionnaire sections III and IV, a VAS was used to assess risk perception scores. Respondents were asked to evaluate their perception of risk of having an ADR for each drug class, and the perceived risk of ADRs was assessed by measuring the distance by the mark made by the participant in the 10 cm visual scale and transforming this to a score ranging between 0 and 10.

A final question was added to classify the perception of the likelihood of potential ADRs for antidiabetic drugs. The selection of these ADRs was based on bibliographic research and included hypoglycaemia,^56–65^ loss of appetite,^62,63,66,67^ increase of appetite,^56^ diarrhea,^56,62,63,68^ nausea,^56,57,62–64,68^ cutaneous reactions,^61,62,64,68^ urinary tract infection,^61,69^ and allergic reactions.^56,62,63,65^

The questionnaire was constructed using layman’s language and everyday wording to define medicines and drug classes and examples of medicines, in order to facilitate its comprehension.^70^

### Data analysis

Descriptive analyses were used to provide an overview of patient characteristics. For comparison of age, educational level and the onset of diabetes of the two groups of people with diabetes, Pearson’s chi-square (χ^2^) was used. This test was also used for the comparison among the groups of attitudes and knowledge regarding pharmacovigilance in Portugal.

For the comparison of opinions and experiences about ADRs and perceived risk for the pre-specified different drug classes, the Kruskal–Wallis test (KW) was used. When the test provided strong evidence of a difference (*p* < 0.05) between the mean ranks of at least one pair of groups, a *post hoc* test using Dunn’s multiple comparison test was carried out for the three pairs of groups, to check evidence of a difference between the two groups (*p* value was set as < 0.05). All *p*-values were two-tailed, with statistical significance set as *p* < 0.05.

In order to analyse risk perception for a different type of diabetes patients related to anti-diabetic drugs, a Pearson’s chi-square (χ^2^) test was performed. For all Pearson’s χ^2^ tests performed, significance was based on a two-sided χ^2^ test and was set at *p* < 0.05. If the expected counts in the contingency table are less than 5, the results from the χ^2^ test are not statistically valid, and Fisher’s exact test was used. The results between type 1 and type 2 diabetes patients were assessed separately and the results are presented in Tables 5 and 6. Data were analysed using statistical SPSS^®^ Statistics Version 22.0 software (IBM Corporation, Armonk, NY, USA).

## Results

### Participation in the study and respondent’s characteristics

A total of 314 patients participated in the study, including 104 patients with diabetes followed in the patient organisation APDP (APDP Group), 106 patients with diabetes not followed in the patient organisation, and 104 patients without diabetes. Table 1 shows the characteristics of patients surveyed, regarding gender, age, level of education, time onset of diagnosis of diabetes and diabetes type.

**Table 1.** Personal characteristics of respondents.

Characteristics	People without diabetes	People with diabetes	
		Not followed at the patient organisation	Followed at the patient organisation
	Total (*n* = 104)	Total (*n* = 106)	Total (*n* = 104)
**Gender (*p* = 0.757)**			
Male	51 (49.0%)	50 (47.2%)	62 (59.6%)
Female	53 (51.0%)	56 (52.8%)	42 (40.4%)
**Age [group (mean value)] (*p* = 0.939)**	60.37 years	Mean 61.06 years	Mean 59.65 years
18–24	1 (1.0%)	1 (0.9%)	1 (1.0%)
25–34	10 (9.6%)	10 (9.4%)	10 (9.6%)
35–44	8 (7.7%)	9 (8.5%)	8 (7.7%)
45–54	17 (16.3%)	19 (17.9%)	16 (15.4%)
55–64	21 (20.2%)	18 (17.0%)	19 (18.3%)
65+	47 (45.2%)	49 (46.2%)	50 (48.1%)
**Educational level (*p* = 0.974)**			
None	4 (3.9%)	4 (3.8%)	3 (2.9%)
Pre-primary, primary, and lower secondary education (levels 0–2)	68 (65.4%)	69 (65.1%)	68 (65.4%)
Upper secondary/post-secondary non-tertiary education (levels 3–4)	18 (17.3%)	19 (17.9%)	19 (18.3%)
First and second stage of tertiary education (levels 5–6)	14 (13.5%)	14 (13.2%)	14 (16.3%)
**Time of diagnosis of diabetes (*p* < 0.01)**			
Less than 12 months	–	4 (3.8%)	6 (5.8%)
1–5 years	–	16 (15.1%)	10 (9.6%)
6–10 years	–	19 (17.9%)	4 (3.8%)
10+ years	–	67 (63.2%)	84 (80.8%)
**Diabetes type (*p* < 0.001)**			
Type 1 diabetes	–	29 (27.4%)	56 (53.8%)
Type 2 diabetes	–	77 (72.6%)	48 (46.2%)

The discrepancy in totals is due to rounding.

Patients were not surveyed about the medicines used in general, except for the medicines used for the control of diabetes, for which the results and perception of risk are detailed in the following, in Table 5.

There were no statistical differences between groups with respect to age, educational level and gender (*p* values were *p* = 0.939, *p* = 0.974 and *p* = 0.757, respectively). Regarding the onset of diabetes, differences were found comparing the two groups of people with diabetes, with the group of patients followed in the patient organisation having a longer time since diagnosis (*p* < 0.01). Type 1 diabetes prevalence presented statistical difference (*p* < 0.001), with 53.8% (*n* = 56) in patients followed in APDP and 27.4% (*n* = 29) in patients not followed at APDP.

Table 2 shows the attitudes and knowledge regarding pharmacovigilance in Portugal among the groups.

**Table 2.** Questions about the attitudes regarding Pharmacovigilance.

Questions	People without diabetes		People with diabetes			
			Not followed at the patient organisation		Followed at the patient organisation	
	Total (*n* = 104)		Total (*n* = 106)		Total (*n* = 104)	
**Did you know that it is possible for patients to spontaneous reporting a possible ADR?**						
Yes	2 (1.9%)		1 (0.9%)		5 (4.8%)	
No	102 (98.1%)		105 (99.1%)		99 (95.2%)	
**Are you aware of the Pharmacovigilance system in Portugal for reporting side effects from medication?**						
Yes	4 (3.9%)		2 (1.9%)		6 (5.8%)	
No	100 (97.1%)		104 (98.1%)		98 (94.2%)	
**Have you ever had side effects from any medicine?**	Yes	No	Yes	No	Yes	No
	20 (19.2%)	84 (80.1%)	27 (25.5%)	79 (76.0%)	40 (38.5%)	64 (61.5%)
**If you had a suspected side effect, what would you do? (more than one answer possible)**	**↓**	**↓**	**↓**	**↓**	**↓**	**↓**
I would talk with my general practitioner/specialist doctor	14 (70.0%)	45 (53.6%)	19 (70.4%)	44 (55.7%)	37 (92.5%)	53 (82.8%)
I would talk to my pharmacist	11 (55.0%)	45 (53.6%)	13 (48.1%)	34 (44.2%)	12 (30.0%)	17 (26.6%)
I would report it to the pharmacovigilance centre	1 (5.0%)	1 (1.2%)	1 (3.7%)	1 (1.3%)	4 (10.0%)	2 (3.1%)
I would stop the medication	1 (5.0%)	4 (4.8%)	–	5 (6.5%)	–	1 (1.6%)
I wouldn’t do anything	–	1 (1.2%)	–	4 (5.2%)	–	1 (1.6%)

The discrepancy in totals is due to rounding.

Most of the patients did not know about the possibility of reporting an ADR (97.5%, *n* = 306), or about the pharmacovigilance system in Portugal (96.2%, *n* = 302). More than a quarter of patients (27.7%, *n* = 87) described that they had experienced an ADR, with patients with diabetes referring more side effects in the past compared with patients without diabetes. Patients followed at APDP reported having experienced an ADR in the past more often than people without diabetes (*p* < 0.01).

Patients who had experienced a side effect in the past had different attitudes regarding suspected ADRs: they would talk to their doctor or pharmacist more often and would report more to the pharmacovigilance centre than patients that had not experienced side effects in the past (Table 2). Overall, patients talk to their doctor (67.5%) or to their pharmacist (45.2%). Reporting an ADR was noted by only 3.2% of respondents as an action to do if they suffered an ADR, while 3.5% mentioned that would stop the medication (*n* = 11) and a few patients [1.9% (*n* = 6)] said they would do nothing.

Groups were also compared regarding their opinions and experiences about ADRs (Table 3). Kruskal–Wallis tests showed some significant differences between groups regarding the statements presented in Table 3.

**Table 3.** Opinions and experiences about ADRs.

Sentences	Group	Strongly disagree	Disagree	Neutral	Agree	Strongly agree	N/A
1 - I would like to have more information about how to report.^b,c^	Diabetics followed at APDP	9 (8.7%)	4 (3.9%)	12 (11.5%)	44 (42.3%)	33 (31.7%)	2 (1.9%)
	Diabetics not followed at APDP	12 (11.3%)	11 (10.4%)	19 (17.9%)	35 (33.0%)	29 (27.4%)	
	Non-Diabetic Patients	27 (26.0%)	19 (18.3%)	18 (17.3%)	27 (26.0%)	13 (12.5%)	
2 - I would like to have more information about the ADRs related to my medicines.^b^	Diabetics followed at APDP	4 (3.9%)	11 (10.6%)	17 (16.4%)	44 (42.3%)	27 (26.0%)	1 (1.0%)
	Diabetics not followed at APDP	13 (12.3%)	11 (10.4%)	22 (20.8%)	27 (25.5%)	31 (29.3%)	2 (1.9%)
	Non-Diabetic Patients	14 (13.5%)	19 (18.3%)	23 (22.1%)	22 (21.2%)	22 (21.2%)	4 (3.9%)
3 - I would like to have more information about general ADRs related to anti-diabetic medication.^a,b,c^	Diabetics followed at APDP	6 (5.8%)	7 (6.7%)	15 (14.4%)	29 (27.9%)	46 (44.2%)	1 (1.0%)
	Diabetics not followed at APDP	11 (10.4%)	12 (11.3%)	28 (26.4%)	37 (34.9%)	18 (17.0%)	
	Non-Diabetic Patients	19 (18.3%)	28 (26.9%)	18 (17.3%)	18 (17.3%)	5 (4.8%)	16 (15.4%)
4 - In the future, I will report an ADR if it is not mentioned in the patient information leaflet.^c^	Diabetics followed at APDP	17 (16.4%)	23 (22.1%)	36 (34.6%)	16 (15.4%)	11 (10.6%)	1 (1.0%)
	Diabetics not followed at APDP	10 (9.4%)	30 (28.3%)	39 (36.8%)	17 (16.0%)	9 (8.5%)	1 (0.9%)
	Non-Diabetic Patients	19 (18.3%)	36 (34.6%)	30 (28.9%)	13 (12.5%)	3 (2.9%)	3 (2.9%)
5 - In the future, I will report an ADR if it is serious.^a,b^	Diabetics followed at APDP	11 (10.6%)	15 (14.4%)	26 (25.0%)	39 (37.5%)	11 (10.6%)	2 (1.9%)
	Diabetics not followed at APDP	29 (27.4%)	23 (21.7%)	25 (23.6%)	26 (24.5%)	3 (2.8%)	–
	Non-Diabetic Patients	22 (21.2%)	20 (19.2%)	30 (28.9%)	24 (23.1%)	7 (6.7%)	1 (1.0%)
6 - In the future, I will report an ADR if it is unexpected.^b^	Diabetics followed at APDP	9 (8.7%)	28 (26.9%)	27 (26.0%)	26 (25.0%)	12 (11.5%)	2 (1.9%)
	Diabetics not followed at APDP	15 (14.2%)	21 (19.8%)	37 (34.9%)	26 (24.5%)	6 (5.7%)	1 (0.9%)
	Non-Diabetic Patients	15 (14.4%)	30 (28.9%)	39 (37.5%)	13 (12.5%)	5 (4.8%)	2 (1.9%)
7 - My doctor explained me the possible ADR of my medication.^a,b^	Diabetics followed at APDP	14 (13.5%)	21 (20.2%)	18 (17.3%)	26 (25.0%)	24 (23.1%)	1 (1.0%)
	Diabetics not followed at APDP	18 (17.0%)	29 (27.4%)	24 (22.6%)	27 (25.5%)	8 (7.6%)	–
	Non-Diabetic Patients	23 (22.1%)	20 (19.2%)	37 (35.6%)	12 (11.5%)	7 (6.7%)	5 (4.8%)
8 - My doctor explained me what to do if I have an ADR related to my medicines.^b^	Diabetics followed at APDP	19 (18.3%)	18 (17.3%)	29 (27.9%)	26 (25.0%)	11 (10.6%)	1 (1.0%)
	Diabetics not followed at APDP	23 (21.7%)	28 (26.4%)	29 (27.4%)	19 (17.9%)	6 (5.7%)	1 (0.9%)
	Non-Diabetic Patients	23 (22.1%)	33 (31.7%)	27 (26.0%)	13 (12.5%)	5 (4.8%)	3 (2.9%)
9 - My doctor explained to me how to report an ADR.	Diabetics followed at APDP	35 (33.7%)	36 (34.6%)	27 (26.0%)	4 (3.9%)	1 (1.0%)	1 (1.0%)
	Diabetics not followed at APDP	25 (23.6%)	42 (39.6%)	32 (30.2%)	3 (2.8%)	4 (3.8%)	
	Non-Diabetic Patients	29 (27.9%)	44 (42.3%)	27 (26.0%)	4 (3.9%)	–	
10 - My pharmacist explained to me the possible ADR of my medication.	Diabetics followed at APDP	26 (25.0%)	36 (34.6%)	20 (19.2%)	17 (16.4%)	3 (2.9%)	2 (1.9%)
	Diabetics not followed at APDP	24 (22.6%)	27 (25.5%)	22 (20.8%)	21 (19.8%)	12 (11.3%)	
	Non-Diabetic Patients	25 (24.0%)	20 (19.2%)	33 (31.7%)	19 (18.3%)	7 (6.7%)	
11 - My pharmacist explained to me what to do if I have an ADR related to my medicines.	Diabetics followed at APDP	30 (28.9%)	34 (32.7%)	26 (25.0%)	8 (7.7%)	2 (1.9%)	4 (3.9%)
	Diabetics not followed at APDP	16 (15.1%)	43 (40.6%)	33 (31.1%)	12 (11.3%)	2 (1.9%)	
	Non-Diabetic Patients	28 (26.9%)	38 (36.5%)	31 (29.8%)	2 (1.9%)	4 (3.9%)	1 (1.0%)
12 - My pharmacist explained to me how to report an ADR.	Diabetics followed at APDP	27 (26.0%)	41 (39.4%)	24 (23.1%)	5 (4.8%)	3 (2.9%)	4 (3.9%)
	Diabetics not followed at APDP	24 (22.6%)	39 (36.8%)	28 (26.4%)	12 (11.3%)	3 (2.8%)	
	Non-Diabetic Patients	37 (35.6%)	28 (26.9%)	23 (22.1%)	10 (9.6%)	6 (5.8%)	

a Significative differences between APDP Group and diabetes Group not under APDP.

b Significative differences between APDP Group and non-diabetics.

c Significative differences between diabetes Group not under APDP and non-diabetics. The discrepancy in totals is due to rounding.

ADR, adverse drug reaction; APDP, Portuguese Diabetes Association.

Both groups of patients with diabetes are more interested in receiving information on how to report ADRs than people without diabetes [KW χ^2^(2) = 31.55, *p* < 0.001, Dunn’s test for both comparisons *p* < 0.001]. Patients followed at APDP tend to have more information about the ADRs related to their medicines, compared with people without diabetes [KW χ^2^(2) = 8.99, *p* = 0.011, Dunn’s test *p* = 0.009]. They also show higher intention to acquire more information about general ADRs related to diabetes medication, compared with both comparison groups. [KW χ^2^(2) = 57.59, *p* < 0.001, Dunn’s test was *p* < 0.001 for both groups]. Patients not followed at the patient organisation will report an ADR if it is not mentioned in the patient information leaflet more often than people without diabetes [KW χ^2^(2) = 7.81, *p* = 0.020, Dunn’s test *p* = 0.031]. Patients followed at APDP will more often report an ADR in the future if it is serious [KW χ^2^(2) = 17.36, *p* < 0.001; Dunn’s test was *p* < 0.05 for both groups], or unexpected [KW χ^2^(2) = 6.45, *p* = 0.040 and Dunn’s test *p* = 0.038 vs people without diabetes]. These patients agreed more that their doctors explained about possible ADRs for the medication [KW χ^2^(2) = 12.76, *p* = 0.002; Dunn’s test was *p* < 0.05 for both groups]. Their doctors also explained them what to do if they have an ADR, when compared with people without diabetes [KW χ^2^(2) = 8.36, *p* = 0.015, Dunn’s test *p* = 0.014].

Table 4 shows the perceived risk of ADRs for different drug groups. Mean VAS scores were presented together with 25th−75th percentiles. The score is presented in a range from 0 to 10.

**Table 4.** The perceived risk of ADRs.

Drug groups (mean scores of the perceived risk of ADRs on visual analogue scales - (25th−75th percentiles)	People without diabetes	People with diabetes	
		Not followed at the patient organisation	Followed at the patient organisation
	Total (*n* = 104)	Total (*n* = 106)	Total (*n* = 104)
Chemotherapy/Cytotoxic drugs	6.9 (5.3–8.8)	7.0 (5.8–8.5)	7.1 (5.6–8.6)
Antibiotics	5.1 (3.6–6.8)	4.4 (2.6–6.3)	5.0 (3.4–6.8)
Insulin	5.0 (3.4–6.7)	5.7 (4.4–7.5)	6.4 (5.1–7.9)^a,b^
Antidepressants	4.7 (2.9–6.3)	5.1 (2.5–7.2)	4.9 (3.2–6.8)
Anti-inflammatory drugs	4.4 (2.3–6.5)	4.0 (2.1–5.7)	4.3 (2.6–5.7)
Drugs for diabetes (in general)	4.3 (2.0–6.4)	4.9 (2.9–6.8)	5.6 (3.6–7.6)^a,b^
Oral hypoglycemic drugs	4.3 (2.3–6.1)	5.2 (3.7–6.9)	5.4 (4.0–6.8)^a,b^
Anxiolitics	4.3 (2.2–6.3)	5.1 (2.4–7.2)	5.2 (2.6–7.2)
Hypocholesterolaemic drugs	4.3 (1.9–6.6)	4.3 (2.6–6.3)	4.1 (2.4–5.3)
Contraceptive pills	4.2 (2.3–5.8)	4.5 (2.4–6.7)	4.1 (2.3–5.8)
Emergency contraception (morning after pill)	4.1 (1.5–6.8)	4.7 (2.5–6.9)	4.3 (2.5–6.6)
Anticoagulants and antithrombotic drugs	4.0 (2.1–5.9)	4.9 (3.4–6.3)^c^	5.1 (3.2–7.0)^b^
Aspirin	4.0 (2.1–6.4)	4.2 (2.0–6.2)	4.4 (2.4–6.4)
Anti-hypertensive drugs	3.8 (1.5–6.8)	4.2 (2.1–6.1)	4.2 (2.8–5.5)
Phytotherapy drugs	3.0 (1.8–4.2)	3.2 (2.0–4.4)	3.7 (1.8–5.4)^b^
Homeopathic drugs	2.7 (1.5–3.8)	2.2 (1.3–3.4)^c^	2.2 (1.3–3.0)^b^
Vitamin supplements	2.5 (1.2–3.7)	2.4 (1.3–3.3)	2.4 (1.2–3.2)

a Significative differences between APDP Group and diabetes Group not under APDP.

b Significative differences between APDP Group and non-diabetics.

c Significative differences between diabetes Group not under APDP and non-diabetics. The discrepancy in totals is due to rounding.

ADR, adverse drug reaction.

Drug classes were ranked according to the mean score of the perceived risk of ADRs obtained in the three groups of patients (Table 4). Kruskal–Wallis tests comparing scores between the three groups were significant for the following drug classes: drugs for diabetes in general, oral hypoglycaemic drugs, insulin, anticoagulants and antithrombotic drugs, phytotherapy drugs and homoeopathic drugs. According to the results presented in Table 4, the overall risk perception is higher among patients with diabetes (both groups) *versus* patients without diabetes. Patients followed at APDP showed an increased risk perception score for medicines related to their disease (anti-diabetic drugs in general, oral anti-diabetic drugs and insulins) than the patients not followed in the patient organisation.

This result is interpreted by the authors as demonstrating that knowledge among APDP members about the specific medicines used in diabetes is better when compared with the other groups of patients surveyed for drugs used in the control of diabetes, as insulin and oral hypoglycemiant drugs. Despite communication between doctors and patients about ADRs related to their medication having not been formally tested, the results in knowledge among APDP patients and the agreement with the sentence ‘My doctor explained me the possible ADR of my medication*’*, with higher agreement among APDP members (Table 3), demonstrates that knowledge among APDP member is higher compared with the other two patient groups, and therefore better risk perception may be present among APDP patients.

For anticoagulants and phytotherapy, patients followed at APDP showed higher risk perception than people without diabetes; however, no differences were found when compared with people with diabetes not followed in the patient organisation. As an example, phytotherapy products can change the effect of certain medicines used in diabetes, and the interactions of antidiabetic drugs and herbs may result in antagonistic or enhancement effects. Some plants like Aloe vera (*Aloe barbadensis sp*.), St. John’s wort (*Hypericum perforatum sp*.) or Ginseng (*Panax ginseng* sp.), which are widely used, could have effects on glucose blood levels and, when combined with drugs for diabetes, could enhance potential interactions. The perception of risk for the use of phytotherapy could be related with the perception that some of these plants could potentially affect the normal effect of medicines used in diabetes, with a better understanding of possible interactions between the medicines used in diabetes and some plants by patients with diabetes. Although it is not expected that patients understand all the interactions between different substances and their drugs for control of diabetes, some information may be reinforced by communication with health professionals (doctors, pharmacists). Once again, communication between HCPs and patients has not been tested and therefore should be the subject of further research.

Table 5 shows the risk perception about medicines used by patients for the control of diabetes. The results presented in the Table 5 refer to the two groups of patients with diabetes only, and compare the consumption and risk perception of diabetic drugs used in the two groups. There are no statistical differences between both groups of patients with diabetes regarding the perceived risk of ADRs related to their diabetes medicines.

**Table 5.** Perception of the risk of diabetic drugs used and ADRs.

Drug groups (mean scores of the perceived risk of ADRs on VAS (25th−75th percentiles)	Patients not followed at the patient organisation (*n* = 106)		Patients followed at the patient organisation (*n* = 104)	
	Type 1 diabetes	Type 2 diabetes	Type 1 diabetes	Type 2 diabetes
Metformin	4.2 (3.5–4.5) *n* = 14	4.4 (3.8–5.2) *n* = 52	4.5 (3.8–5.5) *n* = 29	4.2 (3.5–5.2) *n* = 35
Sulfonylureas	3.4 (3.3–6.0)^a^ *n* = 5	4.0 (3.2–4.3) *n* = 17	3.3 (3.0–4.2) *n* = 10	3.4 (3.2–3.7) *n* = 7
Dipeptidyl peptidase-4 inhibitors	5.7 *n* = 3	4.3 (3.7–5.5)^b^ *n* = 14	4.6 (3.4–5.7) *n* = 6	2.1 (0.4–5.1) *n* = 4
Dipeptidyl peptidase-4 inhibitors + Metformin	4.6 (3.6–5.8) *n* = 12	4.8 (3.8–5.6) *n* = 37	4.4 (3.5–4.9) *n* = 18	4.1 (3.2–5.2) *n* = 19
SGLT-2 inhibitors	4.7^a^ *n* = 3	6.1 (5.6–6.4) *n* = 5	3.8 *n* = 3	5.1 (4.4–6.9) *n* = 9
SGLT-2 inhibitors + Metformin	–	3.9 *n* = 3	5.4 *n* = 3	6.9 *n* = 1
Injectable glucagon-like peptide analogs and agonists	6.8 *n* = 3	7.2 *n* = 2	6.8 (5.3–7.1) *n* = 9	7.0 (5.1–8.2) *n* = 3
Insulin	5.4 (4.7–7.6) *n* = 29	6.5 (4.4–8.0) *n* = 11	5.2 (4.4–7.2) *n* = 54	5.4 (4.9–6.8) *n* = 17

a Differences between Type 1 and Type 2 patients in the same group of patients.

b Differences between the same type of diabetes between the two groups of patients.

ADR, adverse drug reaction; SGLT, sodium-glucose transport protein; VAS, visual analogue scale.

From the data analysed, the medicines most used for the management of diabetes were metformin and insulins, followed by the combination of metformin with dipeptidyl peptidase 4 (DPP-4) inhibitors. Thiazolidinediones, meglitinides and alpha-glucosidase inhibitors were not considered for analysis because they were not used by patients surveyed. Glucagon was not presented in the table because only two patients used it.

The perception of risk of developing a potential ADR related to diabetes medicines was surveyed, and the differences between groups were compared and are listed in Table 6.

**Table 6.** Perception of the relationship between drugs for diabetes and adverse reactions.

Perception of the relationship between drugs for diabetes and adverse reactions (mean score) (25th−75th centiles)	People without diabetes	People with diabetes			
		Not followed at the Patient Organisation Total (*n* = 106)		Followed at the Patient Organisation Total (*n* = 104)	
	Total (*n* = 104)	Type 1 (*n* = 29)	Type 2 (*n* = 77)	Type 1 (*n* = 56)	Type 2 (*n* = 48)
Hypoglycaemia	5.0 (3.0–7.0)	5.9 (4.0–8.0)^a^	5.3 (4.0–7.0)	6.9 (6.0–8.8)	6.2 (4.0–8.0)
Increased appetite	4.9 (2.0–7.0)	4.1 (2.0–6.0)	3.9 (2.0–5.0)	3.1 (1.0–5.0)	3.9 (1.0–5.0)
Decreased appetite	2.9 (1.0–4.0)	5.5 (4.0–7.0)	5.3 (3.0–7.0)	5.7 (4.0–7.0)	5.6 (4.0–7.0)
Diarrhoea	2.6 (1.0–3.0)	5.0 (3.0–7.0)	4.8 (3.0–7.0)	4.3 (2.0–7.0)	4.8 (2.0–7.0)
Nausea	4.5 (2.0–7.0)	5.2 (3.0–8.5)	4.5 (2.0–7.0)	4.8 (3.0–7.0)	4.7 (3.0–7.0)
Skin reactions	3.1 (2.0–4.0)	6.5 (4.5–9.0)^a,b^	3.6 (2.0–5.0)	5.7 (4.0–8.0)^a^	4.4 (4.0–8.0)
Urinary Infections	1.5 (1.0–1.0)	4.5 (3.0–6.0)	3.8 (2.0–6.0)	3.9 (2.0–6.0)	4.0 (2.0–6.0)
Allergic reactions	3.1 (1.0–5.0)	3.9 (2.0–5.5)	3.3 (2.0–5.0)	3.6 (2.0–5.0)	3.8 (2.0–5.0)

a Differences between Type 1 and Type 2 patients in the same group of patients.

b Differences between the same type of diabetes between the two groups of patients.

Both groups of people with diabetes had a higher risk perception score for all symptoms except appetite increase. APDP patients had a significantly higher risk perception for hypoglycaemia then the other groups. For nausea and allergic reactions, no statistical differences were found. Comparison between patients with the same type of diabetes was performed between the two groups of patients; however, since the sample size was too small and because type 1 diabetes was more prevalent in APDP group and type 2 diabetes was more prevalent in the comparison group, it was not possible to obtain reliable results.

## Discussion

The present study was performed to investigate putative differences in the perceived risk of ADRs associated with the use of medicines among patients with diabetes, including those followed in a patient organisation.

### Knowledge and attitudes about ADR reporting

Patients were surveyed about opinions and experiences on pharmacovigilance, including ADR reporting. Patients with diabetes appear to have a higher interest in receiving information about the ADR reporting process, about their general medicines and about the medicines they use for diabetes, leading to an opportunity for patient organisations to carry out educational interventions that can give patients the information they are interested in. In the patients surveyed, all groups presented low knowledge about the opportunity for patients to report possible adverse reactions themselves and low awareness about the existence of a pharmacovigilance system in Portugal for reporting ADRs. Despite this, approximately a quarter of patients surveyed admitted to have suffered an ADR in the past, with patients with diabetes referring to more side effects than patients without diabetes. Patients that are followed at a diabetes patient organisation showed a positive interest in receiving information about how to report compared with the other groups and would like to receive more information about ADRs related to their medicines, including diabetes medication in general. They also more often agreed that their doctor gave them information regarding possible ADRs relating to their medication and also more often explained what to do if they experience an ADR. These results seem to indicate better communication between practitioners and patients followed at APDP compared with patients that were not being followed in the diabetes organisation. No statistically differences were found between any groups in the answers relating to the information received from the pharmacy, which is in accordance with the findings described by Varga *et al*., which stated that only a small number of physicians and pharmacists are taking an active approach in informing patients about the possibility of drug-related damage.^71^ Patients with diabetes showed more positive opinions related to pharmacovigilance. As expected, they would like to have more information about general ADRs related to the anti-diabetic medication. However, they also showed more intention to have information on how to report compared with non-diabetic patients.

### Risk perception for diabetes drugs

Risk perception is usually very variable between patients and is related to their personal experiences. People without diabetes look at the risks of diabetic drugs differently than patients who are using them.

APDP patients had a higher perception of the risk of having ADRs while using medicines in general; however, for most medicine groups, differences were not statistically significant. Patient organisation’ patients more often carry type 1 diabetes and have a longer time since diagnosis, and admitted to having experienced an ADR more often compared with other groups, which could alter their perception of risk regarding their medicines. On the other hand, the fact that they are involved with a patient organisation may influence a higher risk perception of these patients compared with other groups, making them better informed about the risks of medicines due to the fact they were receiving information from APDP and/or from their practitioners during their visits to the diabetes clinic.

Compared with other studies conducted in general patients regarding their risk perception of having ADRs, using the same method of assessment (VAS), patients with diabetes surveyed in this study showed a higher risk perception score for medicines they potentially use in their disease (oral antidiabetics and insulin) than patients included in other studies.^5^ From the perception of the relationship between drugs for diabetes and adverse reactions presented in Table 5, we conclude that patients usually underrate the risk of ADRs of their medications, which is also in accordance with previous studies that assessed knowledge of ADRs in a group of patients.^7^ In another study conducted in Thailand, perception and knowledge concerning medicines risks is generally low, but higher in those who received side effect information.^16^ In accordance with this, patients with diabetes potentially received information related to their medicines during medical appointments, which could explain the higher risk perception in medicines used in diabetes. No studies were found comparing different groups of patients with or without disease and/or being members of a patient’s organisation. As far as the authors know, this is the first study conducted to assess the impact of the disease on risk perception, as well as the impact of being a member of a patient organisation.

In some drug groups, such as sulfonylureas, the results showed a lower risk perception for type 1 patients. Since patients with type 1 diabetes will not receive some oral antihyperglycemic drugs (such as metformin or sulfonylureas), it was expected that they will be less educated about these medicines and presented a lower risk perception when compared with patients with type 2 diabetes. Nevertheless, in other drug groups presented in Table 5, the results are not conclusive due to the small sample size and the fact that type of diabetes differed among the two groups compared (*p* < 0.001), with more patients with type 1 diabetes in the APDP group and more patients with type 2 in the diabetic patients comparison group. Different types of diabetes patients did not show significant differences in risk perception to all drugs used in the control of diabetes, and overall had a higher risk perception for insulin as compared with oral hypoglycaemic agents.

In the comparison between type of diabetes, results mostly seem to reflect the knowledge patients have regarding the medicines they use, which differs for type 1 and type 2 diabetes patients (Tables 5 and 6). Comparison between patients with the same type of diabetes was performed between the two groups of patients; however, since the sample size was too small, it was not possible to obtain reliable results. Also, as expected, differences were found when comparing patients with diabetes *versus* patients without diabetes.

Results show significant differences between groups for most of the specific ADRs surveyed, except nausea and allergic reactions (Table 6). For hypoglycaemia, patients followed at the patient organisation had a significantly higher score of their risk perception compared with patients not followed at APDP. However, this is explained by the pattern of use of antidiabetic medicines by the two groups. The two groups of patients with diabetes have different experience of the disease. Patients followed at APDP represent mostly type 1 patients, which reflects a higher number of insulin users. The impact of their experience is reflected in the side effects perceived: higher perception of side effects like hypoglycaemia is expected and was proved, since this side effect its related with the use of insulin. In the other group, more type 2 patients, who have a higher score of risk perception for most used oral medicines (such as metformin, metformin + DPP-IV inhibitors and sulfonylureas), are present compared with type 1 patients of the same group. Once more these results were expected since type 2 diabetes patients use more oral forms of medication for the control of diabetes.

Although these results may indicate that a patient organisation seems to have an important role in increasing patients’ risk perception, positive attitudes towards pharmacovigilance and that the organisation changes the attitudes and perceptions about the disease and treatment, further studies would be needed to assess the causality of this relationship. In addition to the possible difference in perception related to diabetes type, it could be that particularly those patients with positive attitudes and higher risk perceptions would become a member of a patient organisation. In the future, more qualitative research could be done to investigate this. The authors consider that the findings are not restricted to diabetes patients, so alignment to other disease groups should be investigated.

### Strengths and limitations of the study

One of the strengths of this study is related to the questionnaire construction, which was developed by authors together with a clinical research team associated with a diabetes patient organisation, and their inputs were taken in account to construct the final questionnaire. To construct content validity, volunteers were asked whether topics/questions were missing and to provide comments regarding the format and comprehension of each item.

The involvement of the patient organisation in the construction of the questionnaire improved the understanding of the questionnaire by people with diabetes. The questionnaire was field tested by several volunteers (*n* = 16), not included in the sample, before its implementation. Volunteers were asked whether topics/questions were missing and to provide comments regarding the format and comprehension of each item. Following the review of the comments, items within each construct were amended. During pre-testing, respondents mentioned that a few statements were confusing and these were rewritten to provide easier understanding of the questionnaire. The amendments mostly represented changing words or revising sentence structure to increase item comprehension. Despite this, in the data analysis, the authors could observe that the wording of the questionnaire was still not optimal, as certain participants were confused by the answer options.

The similarity between groups in educational level and age improves the comparability of the results. However, there is a difference in diabetes type and the time since diagnosis between groups. We were not able to exactly match patients on these aspects during the sampling phase. This fact is reflected in some of the answers given by both groups, including the risk perception for adverse reactions that are related to drugs used more in a specific type of diabetes (for example: hypoglycaemia related with insulin use in type 1 patients, with patients with type 1 diabetes having a higher risk perception score for hypoglycaemia related with insulin than type 2 patients). In addition, patients were not surveyed about concomitant diseases or the participation in other patient organisations not related with diabetes. Concerning to the previous experience with ADRs, the results showed that more patients followed at ADPD had experienced an ADR in the past. It is possible that these differences across the groups influence their risk perception, with patients that experienced ADRs in the past having higher risk perception scores than the others.

Finally, the three groups analysed were composed mainly of patients with chronic disease. From inquiries in the ‘no diabetes’ comparison group, it was possible to confirm through pharmacy records that 78.8% (*n* = 82) of patients have at least one chronic disease and have used medication for this for a period longer than 6 months. Chronic diseases included mostly heart disease, hypertension, hypercholesterolemia or respiratory diseases, among others. This highlights the validity of this study findings, since patient groups comprised patients with diabetes or other chronic diseases, allowing us to reduce the bias that could be caused by comparing chronic diabetes patients without chronic diseases.

The study also has some other drawbacks: the use of face-to-face survey could lead to social desirability bias, leading to biased answers.^72^ The wording of questions may also influence the way patients responded, causing response bias due to misinterpretation of questions. Selection bias could also be present, since proper randomisation was not used in selecting respondents, due mainly to the selection method of sampling (convenience sample), but also because comparison groups were collected from a different setting than the APDP group, so validity of the results might be threatened and lead to inconclusive and non-comparable results.^73,74^ Propensity scores could not be used as a result of the sampling method since the interpretation is potentially impacted by self-selection and non-randomisation.^75,76^

## Conclusion

Patients with diabetes showed more positive opinions related to pharmacovigilance. They would like to obtain more information about ADRs related to their medication and a higher intention to acquire information on how to report when compared with non-diabetic patients. Patients followed in a diabetes patient association presented a higher score of risk perception, which could be influenced by the presence of the diabetes disease in the patients’ life, as well as by their previous experiences using the medicines, but also by the information received from the patient organisation. The two groups of patients with diabetes have different experiences of the disease, but both present higher perception of side effects related with medicines they use in their respective type of diabetes. Being a member of a patient organisation seems to play an important role in increasing risk perception since they presented the highest risk perception scores for medicines related to their disease. Those patients affirmed that their doctor explained the possible ADRs of their medication and they have higher intention to report ADRs in the future if these are serious or unexpected. Hence, patient organisations are well positioned to be a source from where patients can obtain reliable information, changing their attitudes and perceptions about disease and drug treatments.
